# Dr. Charles S. Jones

**Published:** 1899-09

**Authors:** 


### OBITUARY.

### Dr. Charles S. Jones.

Dr. Charles S. Jones, a well-known dentist, died suddenly at his residence, No. 4116 Spruce Street, Philadelphia, on Sunday, August 20, 1899, of heart failure superinduced by a severe and long-continued attack of gastric fever.

Dr. Jones was born in Philadelphia in April, 1828. He had been connected with dentistry for more than forty years. About 1857 he entered the employ of Samuel S. White, and for a number of years had charge of the domestic sales of porcelain teeth. While so engaged he matriculated at the Philadelphia Dental College, from which he was graduated in 1875. He immediately began the practice of dentistry, in which he was successful, continuing his work until disabled by the illness which finally proved fatal. Dr. Jones was a skillful practitioner, more especially in the prosthetic branch. He was for several years demonstrator of mechanical dentistry in the Dental Department of the University of Pennsylvania, in which position he acquitted himself with satisfaction to the authorities of the college. He took great interest in Masonic work, and was at the time of his death Worshipful Master of Philadelphia Lodge No. 72, F. and A. M.

On the outbreak of the Civil War he entered the army as a lieutenant in the Forty-third Pennsylvania Volunteers, and was promoted to captain.

Dr. Jones was never married. He was a man of quiet, retiring disposition, who preferred always to let his work speak for him; a conscientious man, to whom what he believed was right pointed out the duty to be done. These qualities, combined with his undoubted skill, gathered about him a clientele who believed in him, and formed a part of the circle of personal friends who will sincerely regret his death.

